# Dose reduction potential in cone-beam CT imaging of upper extremity joints with a twin robotic x-ray system

**DOI:** 10.1038/s41598-021-99748-1

**Published:** 2021-10-11

**Authors:** Karsten Sebastian Luetkens, Süleyman Ergün, Henner Huflage, Andreas Steven Kunz, Carsten Herbert Gietzen, Nora Conrads, Lenhard Pennig, Lukas Goertz, Thorsten Alexander Bley, Tobias Gassenmaier, Jan-Peter Grunz

**Affiliations:** 1 Department of Diagnostic and Interventional Radiology, University Hospital Würzburg, Oberdürrbacher Straße 6, 97080 Würzburg, Germany; 2 Institute of Anatomy and Cell Biology, University of Würzburg, Koellikerstraße 6, 97070 Würzburg, Germany; 3 Institute for Diagnostic and Interventional Radiology, Faculty of Medicine and University Hospital Cologne, University of Cologne, Kerpener Straße 62, 50937 Cologne, Germany

**Keywords:** Medical research, Preclinical research

## Abstract

Cone-beam computed tomography is a powerful tool for 3D imaging of the appendicular skeleton, facilitating detailed visualization of bone microarchitecture. This study evaluated various combinations of acquisition and reconstruction parameters for the cone-beam CT mode of a twin robotic x-ray system in cadaveric wrist and elbow scans, aiming to define the best possible trade-off between image quality and radiation dose. Images were acquired with different combinations of tube voltage and tube current–time product, resulting in five scan protocols with varying volume CT dose indices: full-dose (FD; 17.4 mGy), low-dose (LD; 4.5 mGy), ultra-low-dose (ULD; 1.15 mGy), modulated low-dose (mLD; 0.6 mGy) and modulated ultra-low-dose (mULD; 0.29 mGy). Each set of projection data was reconstructed with three convolution kernels (very sharp [Ur77], sharp [Br69], intermediate [Br62]). Five radiologists subjectively assessed the image quality of cortical bone, cancellous bone and soft tissue using seven-point scales. Irrespective of the reconstruction kernel, overall image quality of every FD, LD and ULD scan was deemed suitable for diagnostic use in contrast to mLD (very sharp/sharp/intermediate: 60/55/70%) and mULD (0/3/5%). Superior depiction of cortical and cancellous bone was achieved in FD_Ur77_ and LD_Ur77_ examinations (*p* < 0.001) with LD_Ur77_ scans also providing favorable bone visualization compared to FD_Br69_ and FD_Br62_ (*p* < 0.001). Fleiss’ kappa was 0.618 (0.594–0.641; *p* < 0.001), indicating substantial interrater reliability. In this study, we demonstrate that considerable dose reduction can be realized while maintaining diagnostic image quality in upper extremity joint scans with the cone-beam CT mode of a twin robotic x-ray system. Application of sharper convolution kernels for image reconstruction facilitates superior display of bone microarchitecture.

## Introduction

Fractures of the upper extremity are associated with individual physical impairment and often high socioeconomic relevance. While distal radius fractures are among the most frequent trauma consequences in any emergency department with particularly high incidence in elderly patients^1–3^, dislocations and fractures in the elbow region are more common in younger populations^4^. Plain radiographs usually represent the primary imaging method if traumatic injuries of the upper limb are suspected^5^. Radiograms offer a compromise between fast scan time, ubiquitous availability, and low radiation dose on the one side, and limited 2D image information on the other side^6^. Considering that trauma mechanisms can be vastly heterogeneous, ranging from simple falls on the outstretched hand from standing height to high-velocity injuries in sports activities and motor vehicle accidents^2,7^, 2D imaging may be insufficient for trauma evaluation in some patients. Especially more severe injuries, e.g., multi-fragmentary, intraarticular and displaced fractures of the wrist and elbow require additional 3D assessment for presurgical planning^8^ and image fusion with intraoperative fluoroscopy^9,10^. In addition, computed tomography scans allow for reliable exclusion or detection of radiographically occult fractures in case of divergent clinical presentation^11^. Even for certain chronic conditions or postoperative follow-up imaging, CT can provide valuable diagnostic information with the typical drawback of increased radiation dose^12,13^.

Despite the predominance of multidetector CT in most radiology departments, the value of another technical approach to computed tomography is increasingly recognized for skeletal imaging tasks: Combining a flat-panel detector build with pyramid-shaped beam geometry, cone-beam computed tomography (CBCT) has become a powerful tool for 3D imaging in trauma patients. Detailed visualization of bone microarchitecture^14^, superior positioning options and potential for reduced radiation dose are among the major advantages compared to conventional multidetector CT^15^. Having been considered an integral part of maxillofacial imaging for a long time^16–19^, access to dedicated scanners for the appendicular skeleton has led to increasing relevance of CBCT in musculoskeletal imaging during the last decade^20^.

The twin robotic x-ray system in this study represents a novel approach to the CBCT formula that uses two telescopic arms for 2D and 3D image acquisition instead of a conventional gantry-based approach. Whilst the benefits of the CBCT option for fracture detection have previously been analyzed, data regarding the actual dose reduction potential of this multi-use system is lacking. Therefore, in this work, various combinations of acquisition and reconstruction parameters were evaluated for the CBCT mode of the twin robotic x-ray system in cadaveric wrist and elbow scans, aiming to define the best possible trade-off between image quality and radiation dose.

## Material and methods

### Cadaveric phantoms

**Figure 1:**
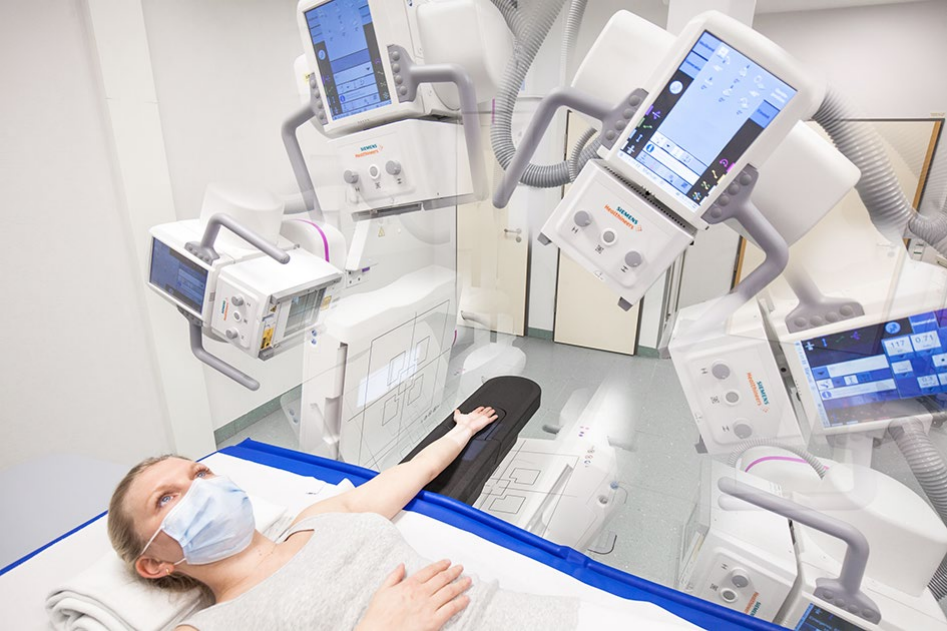
Rendering of staff member demonstrating the scan position for 3D cone-beam CT imaging of upper extremity joints using the tableside trajectory of the twin robotic x-ray system.

For this experimental study, we received permission from the institutional review board of the University of Würzburg, Germany (IRB number 20200506 01). Two formalin-fixed cadaveric specimens were obtained from the anatomical institute of the local university. Body donors had voluntarily donated their bodies to the anatomical institute for study and scientific purposes. Thus, the ethics committee of the University of Würzburg waived additional written informed consent. Bilateral wrist and elbow examinations were performed for both cadaveric specimens in supine position using the twin robotic x-ray system’s tableside scan trajectory. Scanning process for upper extremity imaging is displayed in Fig. 1 by a staff member (informed consent for publication was obtained). All procedures were conducted in accordance with the relevant guidelines and regulations.

### Cone-beam CT scans

For image acquisition, the multifunctional radiography system (Multitom Rax, Siemens Healthcare GmbH, Erlangen, Germany) uses two telescopic arms connected to ceiling rails within the x-ray suite. One of the arms holds a quadratic flat-panel detector (edge length 426 mm), while the other carries the x-ray tube. Different motion trajectories for both arms allow for 2D and 3D imaging in specified positions. CBCT scans are performed with an asymmetric source-to-image distance of 1150 mm, an input field of 213 × 213 mm^2^ and a 2D matrix of 1440 × 1440 un-binned pixels in high-resolution mode, which results in an effective pixel size of 149 µm. The current software version VF 11 (Siemens Healthcare GmbH) supports acquisition of 26 projection images per second with a sweep angle of 200 degrees, hence resulting in a total scan time of 14 s.

Before each CBCT scan, system settings can be manually adjusted to achieve target dose levels. Using a clinically established full-dose protocol (FD) as reference, four other scan protocols with lower radiation doses were evaluated in this study. Images were either acquired with different fixed combinations of tube voltage and tube current–time product (low-dose [LD], ultra-low-dose [ULD]) or with the scanner’s automatic dose modulation feature (modulated low-dose [mLD], modulated ultra-low-dose [mULD]): In this scan mode, a sensor implemented in the flat-panel detector measures the incoming radiation for each projection image. Dependent on the previously determined dose level, the tube current–time product is then adjusted continuously throughout the acquisition process. Scan parameters and corresponding radiation dose values associated with each CBCT examination protocol are summarized in Table 1. Dose-area products for all examinations were obtained from the automatically created scan report. To calculate volume CT dose indices (CTDI_vol_), multiplication by a linear scaling factor that we computed in advance for every combination of tube current–time product and tube voltage was needed. Therefore, dose-length product measurements were performed in the five chambers of a conventional 16 cm PMMA dosimetry phantom (IEC 60601-2-44:2009 compliant). After standard weighting schemes were applied to obtain volume dose-length product values (DLP_vol(16 cm)_), these values were divided by the field of view in z-direction (equal to the beam width) to compute the CTDI_vol(16 cm)_. Finally, dividing the CTDI_vol(16 cm)_ by the DAP resulted in the required scaling factor.

**Table 1:** Scan protocols and radiation dose.

Multitom Rax^a^	Full-dose mode	Low-dose mode	Ultra-low-dose mode	Modulated low-dose mode	Modulated ultra-low-dose mode
Reference kVp	80	80	60	80	80
Scan mAs	2.5	0.6	0.5	Regulated	Regulated
DAP (dGy × cm^2^)	54.3	14.0	3.5	Mean 1.9	Mean 0.9
CTDI_vol(16 cm)_ (mGy)	17.4	4.5	1.2	Mean 0.6	Mean 0.3
Effective dose (µSv)	13.2	3.4	0.9	Mean 0.5	Mean 0.2
Relative dose	100%	25.8%	6.8%	3.8%	1.5%

Operator settings and dose estimation for 3D cone-beam CT examinations with different acquisition protocols.

*DAP* dose-area product, *CTDI*_*vol(16 cm)*_ volume computed tomography dose index (for 16 cm diameter PMMA dosimetry phantom).

^a^Siemens Healthcare GmbH; Erlangen, Germany.

### Reconstruction kernels

Each set of projection data was reconstructed with dedicated 3D processing software (syngo via, Siemens Healthcare GmbH) using different modulation transfer functions characterized by standard convolution kernels: very sharp (Ur77): ρ_10_ = 25.4 lp/cm, ρ_50_ = 16.7 lp/cm; sharp (Br69): ρ_10_ = 15.1 lp/cm, ρ_50_ = 12.3 lp/cm; intermediate (Br62): ρ_10_ = 11.3 lp/cm, ρ_50_ = 9.2 lp/cm (all Siemens Healthcare GmbH). Reformatting was performed in orthogonal planes (axial, coronal and sagittal) with slice thickness of 1.0 mm, increment of 0.5 mm, image matrix of 1024 × 1024 pixels and field of view of 80 mm. Window settings were 3000 and 1000 Hounsfield units (width and center) by default with observers being allowed to modify the contrast manually on screen for their reads.

### Image analysis

After image reconstruction, all datasets were analyzed in randomized and blinded fashion with standard picture archiving and communication software (Merlin, Phoenix-PACS, Freiburg im Breisgau, Germany) by five independent radiologists with 8, 7, 6, 5 and 4 years of experience in musculoskeletal imaging. First, each observer was tasked to evaluate whether the reviewed scan was sufficient for diagnostic use. Second, readers assessed overall image quality and gave dedicated image quality ratings for cortical bone, cancellous bone and soft tissue using a seven-point Likert scale (7 = excellent, 6 = very good, 5 = good, 4 = satisfactory, 3 = fair, 2 = poor, 1 = very poor image quality).

### Statistics

All tests and analyses were conducted using specialized software (SPSS Statistics Version 27 for Mac, IBM, Armonk, New York, USA) with *p* values less than 0.05 indicating statistical significance. Categorical items are reported in form of percentages, frequencies and median values. Wilcoxon signed rank and Friedman tests were carried out to compare two or more paired non-parametric variables. Interrater reliability was assessed by calculation of Fleiss’ kappa with interpretation of agreement following Landis and Koch (1.00–0.81 = almost perfect; 0.80–0.61 = substantial; 0.60–0.41 = moderate; 0.40–0.21 = fair; 0.20–0.00 = slight; < 0.00 = poor agreement)^21^.

## Results

### Dose comparison between scan protocols

Operating with fixed tube current–time products, CTDI_vol(16 cm)_ of low-dose and ultra-low-dose scans was 4.5 and 1.2 mGy, corresponding to 74.2% and 93.1% of dose reduction compared to the standard full-dose scan protocol used in clinical routine (17.4 mGy). With automatic dose regulation activated, modulated low-dose (mean CTDI_vol(16 cm)_ 0.6 mGy) and modulated ultra-low-dose acquisition (mean CTDI_vol(16 cm)_ 0.3 mGy) allowed for dose reduction of 96.6% and 98.3% (*p* < 0.001). Scan parameters and dose values were equal for wrist and elbow scans.

### Image quality assessment

**Figure 2:**
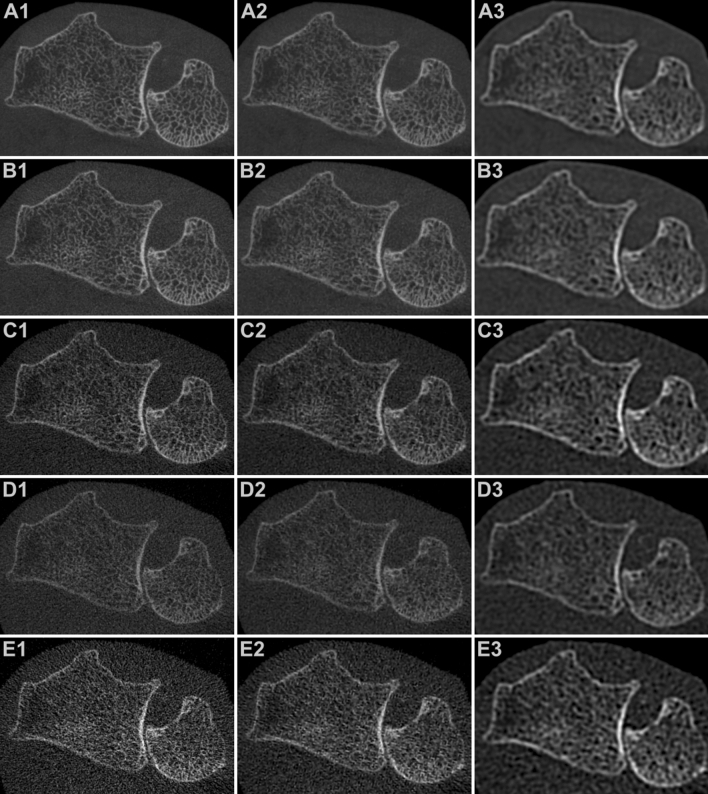
Axial reformatting of 3D wrist scans with varying acquisition parameters and reconstruction kernels: (**A**) Full-dose mode (A1: very sharp; A2: sharp; A3: intermediate). (**B**) Low-dose mode (B1: very sharp; B2: sharp; B3: intermediate). (**C**) Ultra-low-dose mode (C1: very sharp; C2: sharp; C3: intermediate). (**D**) Modulated low-dose mode (D1: very sharp; D2: sharp; D3: intermediate). (**E**) Modulated ultra-low-dose mode (E1: very sharp; E2: sharp; E3: intermediate).

**Figure 3:**
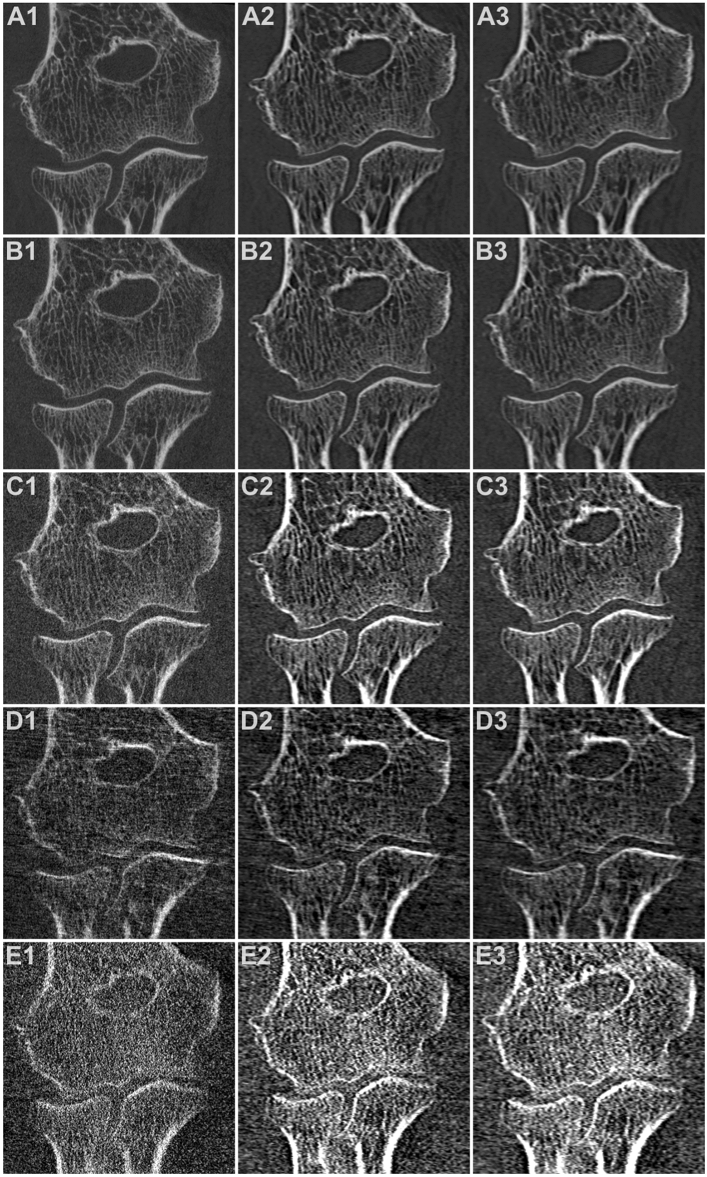
Coronal reformatting of 3D elbow scans with varying acquisition parameters and reconstruction kernels: (**A**) Full-dose mode (A1: very sharp; A2: sharp; A3: intermediate). (**B**) Low-dose mode (B1: very sharp; B2: sharp; B3: intermediate). (**C**) Ultra-low-dose mode (C1: very sharp; C2: sharp; C3: intermediate). (**D**) Modulated low-dose mode (D1: very sharp; D2: sharp; D3: intermediate). (**E**) Modulated ultra-low-dose mode (E1: very sharp; E2: sharp; E3: intermediate).

Independent of the convolution kernel used for image reconstruction, overall image quality of every FD, LD and ULD scan in this study was deemed suitable for diagnostic evaluation in clinical routine by all five radiologists. In contrast, readers considered 40/45/30% of mLD and 100/97/95% of mULD (Ur77/Br69/Br62 kernel) insufficient for clinical imaging. Comparing reconstructions with the same kernel, studies with higher dose levels produced superior overall image quality in upper extremity scans (all *p* < 0.001). Detailed observer ratings are outlined in Table 2. The best overall image quality was reported for FD_Ur77_ examinations (median scale value 7). Evaluation of LD_Ur77_ studies delivered favorable results compared to FD_Br62_ (*p* < 0.001) and comparable image quality to FD_Br69_ scans (*p* = 0.157). No distinction was stated between LD_Br69_ and FD_Br62_ (*p* = 0.475). For examinations with the ULD scan protocol, at least satisfactory image quality (scale value ≥ 4) was declared in 50/58/40% of studies with no significant difference between image processing algorithms (median 3.5/4/3; *p* = 0.072). Fleiss’ kappa was 0.618 (95% confidence interval 0.594–0.641; *p* < 0.001), indicating substantial interrater reliability for image quality assessment. Figures 2 and 3 contain representative CBCT images in axial and coronal orientation for visualization of image quality.

**Table 2:** Overall image quality assessment.

	Very sharp	Sharp	Intermediate
**Overall image quality**			
Full-dose mode	7 (1.00)	6 (1.00)	5 (1.00)
Low-dose mode	6 (1.00)	5 (1.00)	4 (1.00)
Ultra-low-dose mode	3.5 (0.50)	4 (0.58)	3 (0.40)
Modulated low-dose mode	2 (0.13)	2 (0.15)	3 (0.10)
Modulated ultra-low-dose mode	1 (0)	1 (0)	1.5 (0)
**Diagnostic image quality**			
Full-dose mode	100%	100%	100%
Low-dose mode	100%	100%	100%
Ultra-low-dose mode	100%	100%	100%
Modulated low-dose mode	60%	55%	70%
Modulated ultra-low-dose mode	0%	3%	5%

Synopsis of overall image quality evaluation in cadaveric wrist and elbow scans by five radiologists for each combination of convolution kernel and dose protocol. Median rating values and share of examinations with at least “satisfactory” image quality (score ≥ 4) are reported. Percentage of studies that were deemed “suitable for diagnostic use in patient studies” is displayed.

**Figure 4:**
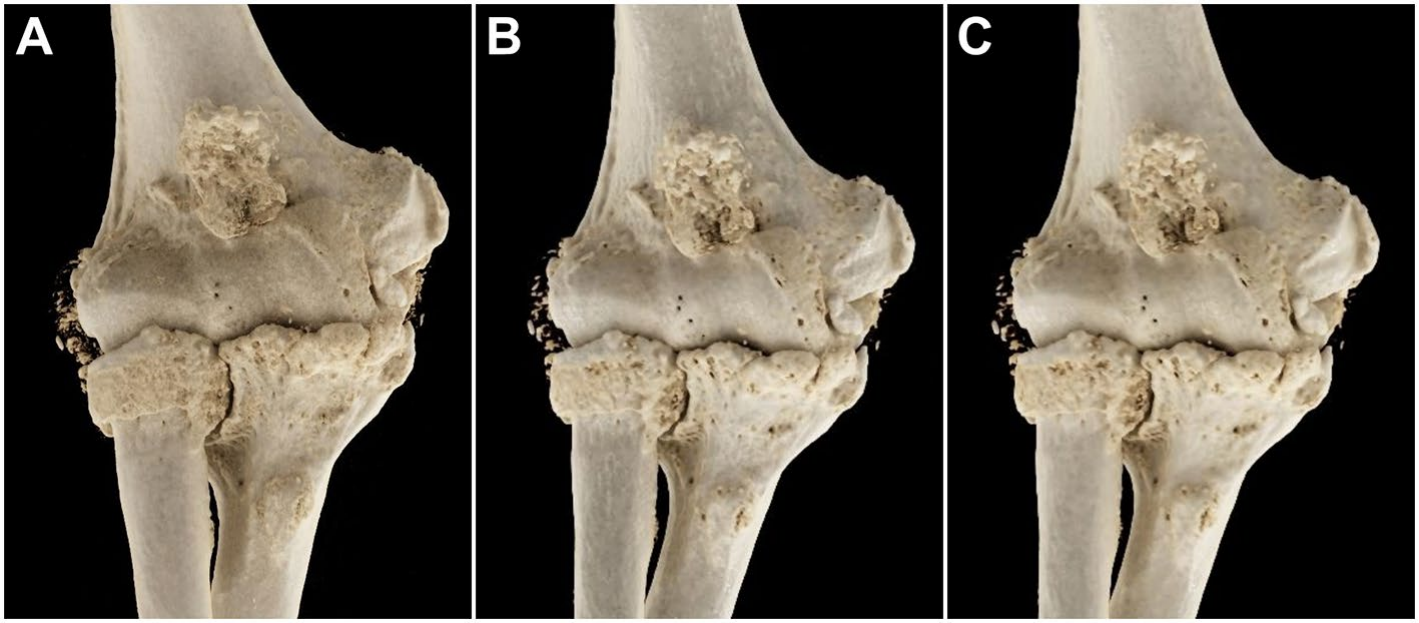
Cinematic volume rendering technique (syngo.via, Siemens Healthcare GmbH) demonstrates the difference in image sharpness provided by the three convolution kernels for full-dose elbow studies: (**A**) Very sharp kernel (Ur77). (**B**) Sharp kernel (Br69). (**C**) Intermediate kernel (Br62).

Comparing the effect of each convolution kernel on the display of bone microarchitecture, visualization of cortical and cancellous bone was best in very sharp reconstructions of full-dose and low-dose examinations (*p* < 0.001), with LD_Ur77_ scans also providing favorable bone depiction compared to FD_Br69_ and FD_Br62_ (*p* < 0.001). Accordingly, sharper ULD_Ur77_ (*p* = 0.005) and ULD_Br69_ (*p* = 0.009) reconstructions were deemed more suitable for differentiation of trabecula and fatty marrow than softer ULD_Br62_ studies. The effect of the different convolution kernels on image sharpness is exemplified in Fig. 4. Quality of cortex display in ultra-low-dose scans was independent of image processing (*p* = 0.764). For soft tissue analysis, no difference between kernels was identified in full-dose (*p* = 0.302) and low-dose image acquisition (*p* = 0.129). In ultra-low-dose studies, both ULD_Br69_ (*p* = 0.008) and ULD_Br62_ (*p* < 0.001) provided superior soft tissue assessability compared to ULD_Ur77_ with no difference between the two softer kernels (*p* = 0.739). Detailed image quality evaluation for bone and soft tissue is summarized in Table 3.

**Table 3:** Detailed image quality assessment for bone and soft tissue.

	Very sharp	Sharp	Intermediate
**Cortical bone**			
Full-dose mode	7 (1.00)	6 (1.00)	5 (1.00)
Low-dose mode	7 (1.00)	6 (1.00)	5 (1.00)
Ultra-low-dose mode	4 (0.50)	4 (0.53)	4 (0.53)
Modulated low-dose mode	3 (0.15)	3 (0.20)	3 (0.35)
Modulated ultra-low-dose mode	1 (0)	1 (0)	1.5 (0)
**Cancellous bone**			
Full-dose mode	7 (1.00)	6 (1.00)	5 (1.00)
Low-dose mode	6 (1.00)	5.5 (1.00)	4 (1.00)
Ultra-low-dose mode	3.5 (0.50)	4 (0.60)	3 (0.35)
Modulated low-dose mode	2 (0.13)	2 (0.10)	3 (0.05)
Modulated ultra-low-dose mode	1 (0)	1 (0)	1 (0)
**Soft tissue**			
Full-dose mode	5 (0.80)	4 (0.80)	4 (0.80)
Low-dose mode	4 (0.65)	4 (0.75)	4 (0.65)
Ultra-low-dose mode	2 (0)	2 (0)	2 (0)
Modulated low-dose mode	1 (0)	1 (0)	1.5 (0)
Modulated ultra-low-dose mode	1 (0)	1 (0)	1 (0)

Dedicated image quality evaluation of cortical bone, cancellous bone and soft tissue by five radiologists for each combination of convolution kernel and dose protocol in cadaveric wrist and elbow scans. Median rating values and share of examinations with at least “satisfactory” image quality (score ≥ 4) are reported.

## Discussion

In this multi-observer study, we evaluated the image quality provided by 15 combinations of acquisition protocols and reconstruction parameters for the CBCT scan mode of a twin robotic x-ray system in cadaveric wrist and elbow imaging. We were able to show that dose reduction of more than 90% is feasible for certain imaging tasks, while maintaining diagnostic image quality with a dedicated ultra-low-dose scan protocol. Reducing the radiation dose even further by implementing detector-based dose modulation resulted in a considerable amount of non-diagnostic studies. Therefore, we postulate that the ultra-low-dose protocol with reduced tube voltage represents the best trade-off between required dose and image assessability for upper extremity scans.

A recent meta-analysis by Nardi et al. identified an average dose of 7.1 µSv for CBCT scans of the appendicular skeleton, stating considerable dose reduction potential in comparison to multidetector CT imaging^22^. Compared to conventional radiography, however, radiation dose of CBCT remains significantly higher in most studies^23^. While scan protocols without a focus on dose reduction may have the potential to overcome the image quality of state-of-the-art multidetector CT scanners, we believe that CBCT can also challenge radiography as the primary method of fracture diagnosis^24^. Due to distance to the radiation-sensitive body trunk, effective dose of extremity imaging is generally lower than for other body regions^25^. Providing diagnostic 3D image information with approximately four times the effective dose of standard 2D radiograms, the ultra-low-dose protocol in this study appears promising for primary use in particular imaging tasks, e.g., carpal bone^14,23^ or radial head evaluation^26^. Furthermore, pediatric radiology could benefit from the reduced radiation dose in cases that require 3D assessment to ascertain the injury pattern, e.g., in distal humerus fractures^26^. To investigate which scan protocol is appropriate for different pathologies should be part of future research. From a technical point of view, the maintained image quality in ultra-low-dose scans may foremost be attributed to the small effective pixel size of 149 µm realized by the combination of asymmetric acquisition geometry (the detector rotates in a smaller radius around the isocenter than the x-ray tube to balance the effect of the focal spot size) and un-binned readout of the flat-panel detector in high-resolution mode.

With very sharp reconstructions of low-dose examinations providing favorable image quality over full-dose scans with softer reformatting, additional dose reduction potential can be realized through image postprocessing. In particular, the superior display of cancellous bone in studies reconstructed with the two sharper convolution kernels may aid fracture assessment due to better discrimination of trabecula and fatty bone marrow. In contrast, the inferior visualization of bone microarchitecture provided by the intermediate convolution kernel might be attributed to the fact that the realized modulation transfer function of this kernel is far below the system’s resolution limit without a defined kernel.

### Limitations

The two formalin-fixed cadaveric specimens were selected without knowledge on body donor age, time of fixation and bone density of the examined wrist and elbow region. Thus, preexisting osteopenia and the demineralizing effect of formalin on bone tissue could have had a negative effect on subjective image quality ratings^27,28^. With examinations in this study limited to cadaveric limbs, the potential effect of motion artifacts in future patient scans could not be estimated. Given the limited number of projection images acquired per scan, the influence of off-center positioning on image quality ought to be evaluated in further studies. However, with motion compensation algorithms continuously improving and comfortable positioning being facilitated by the open scanner architecture, the authors do not expect a considerable loss of image quality in a realistic clinical scenario. Finally, despite being blinded to scan and reconstruction parameters, radiologists could have gained a certain amount of involuntary training during their reading sessions.

## Conclusion

With the cone-beam CT scan mode of a twin robotic x-ray system, dose reduction of more than 90% can be realized for certain upper extremity joint examinations, while maintaining diagnostic image quality. Application of sharper convolution kernels for image reconstruction facilitates superior display of bone microarchitecture, potentially aiding in detection of microfractures in future patient studies.

## Abbreviations

CBCT: Cone-beam computed tomography
CTDI_vol_: Volume computed tomography dose index (mGy)
DAP: Dose-area product
FD: Full-dose scan protocol
LD: Low-dose scan protocol
ULD: Ultra-low-dose scan protocol
mLD: Modulated low-dose scan protocol
mULD: Modulated ultra-low-dose scan protocol
